# Crystal structure of 1-[2-(2,6-di­chloro­phen­yl)-4,5-diphenyl-1*H*-imidazol-1-yl]propan-2-ol

**DOI:** 10.1107/S2056989015006763

**Published:** 2015-04-09

**Authors:** Mehmet Akkurt, Jerry P. Jasinski, Shaaban K. Mohamed, Adel A. Marzouk, Mustafa R. Albayati

**Affiliations:** a Department of Physics, Faculty of Sciences, Erciyes University, 38039 Kayseri, Turkey; b Department of Chemistry, Keene State College, 229 Main Street, Keene, NH 03435-2001, USA; c Chemistry and Environmental Division, Manchester Metropolitan University, Manchester M1 5GD, England; d Chemistry Department, Faculty of Science, Minia University, 61519 El-Minia, Egypt; e Pharmaceutical Chemistry Department, Faculty of Pharmacy, Al Azhar University, 71515 Assiut, Egypt; f Kirkuk University, College of Science, Department of Chemistry, Kirkuk, Iraq

**Keywords:** crystal structure, 1-[2-(2,6-di­chloro­phen­yl)-4,5-diphenyl-1*H*-imidazol-1-yl]propan-2-ol, imidazole ring, amino alcohol, hydrogen bonding, C—H⋯π inter­actions

## Abstract

The central imidazole ring of the title compound, C_24_H_20_Cl_2_N_2_O, is twisted with respect to with the planes of the 2,6-di­chloro­benzene and two phenyl rings, making dihedral angles of 74.06 (18), 28.52 (17) and 67.65 (18)°, respectively. The phenyl ring not adjacent to the N-bonded 2-hy­droxy­propyl group shows the greatest twist, presumably to minimize steric inter­actions. In the crystal, mol­ecules are linked by O—H⋯N and C—H⋯O hydrogen-bond contacts into chains along the *a*-axis direction. The series of parallel chains form a two-dimensional sheet approximately parallel to the *bc* diagonal. In addition, C—H⋯π inter­actions are observed between the sheets. The atoms of the 2-hy­droxy­propyl group and the N atom of the 1*H*-imidazole ring to which it is bonded are disordered over two sets of sites, with an occupancy ratio of 0.722 (5):0.278 (5). The structure was refined as an inversion twin.

## Related literature   

For similar structures and background to the biological properties of imidazole derivatives, see: Mohamed *et al.* (2012 ▸, 2013*a* ▸,*b* ▸); Akkurt *et al.* (2013 ▸); Jasinski *et al.* (2015 ▸).
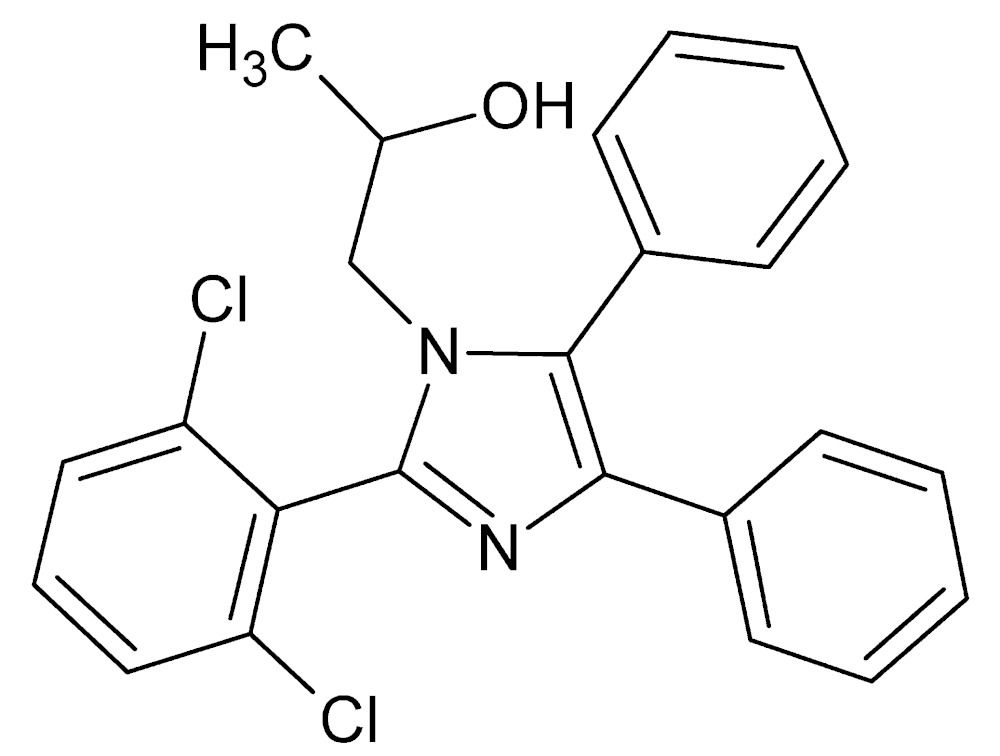


## Experimental   

### Crystal data  

C_24_H_20_Cl_2_N_2_O
*M* *_r_* = 423.32
Orthorhombic, 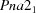

*a* = 12.1468 (4) Å
*b* = 8.4194 (2) Å
*c* = 20.9636 (7) Å
*V* = 2143.92 (11) Å^3^
*Z* = 4
Cu *K*α radiation
μ = 2.86 mm^−1^
*T* = 173 K
0.48 × 0.44 × 0.26 mm

### Data collection  

Agilent Eos Gemini diffractometer
Absorption correction: multi-scan (*CrysAlis PRO*; Agilent, 2014 ▸) *T*_min_ = 0.541, *T*_max_ = 1.000
16532 measured reflections
4076 independent reflections
3888 reflections with *I* > 2σ(*I*)
*R*_int_ = 0.033

### Refinement  

*R*[*F* ^2^ > 2σ(*F* ^2^)] = 0.037
*wR*(*F* ^2^) = 0.097
*S* = 1.05
4076 reflections
278 parameters
15 restraints
H-atom parameters constrained
Δρ_max_ = 0.26 e Å^−3^
Δρ_min_ = −0.18 e Å^−3^
Absolute structure: refined as an inversion twin
Absolute structure parameter: 0.068 (18)

### 

Data collection: *CrysAlis PRO* (Agilent, 2014 ▸); cell refinement: *CrysAlis PRO*; data reduction: *CrysAlis PRO*; program(s) used to solve structure: *SHELXT* (Sheldrick, 2015*a* ▸); program(s) used to refine structure: *SHELXL2014* (Sheldrick, 2015*b* ▸); molecular graphics: *ORTEP-3 for Windows* (Farrugia, 2012 ▸); software used to prepare material for publication: *PLATON* (Spek, 2009 ▸).

## Supplementary Material

Crystal structure: contains datablock(s) global, I. DOI: 10.1107/S2056989015006763/sj5450sup1.cif

Structure factors: contains datablock(s) I. DOI: 10.1107/S2056989015006763/sj5450Isup2.hkl

Supporting information file. DOI: 10.1107/S2056989015006763/sj5450Isup3.cml

**Fig. 1.**
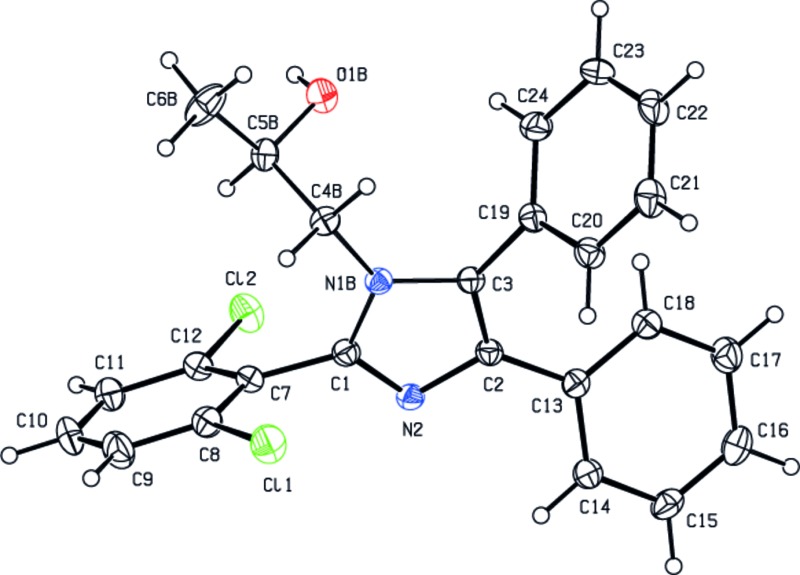
Perspective view of the title molecule with 30% probability displacement ellipsoids. Only the major disorder component is shown.

**Fig. 2.**
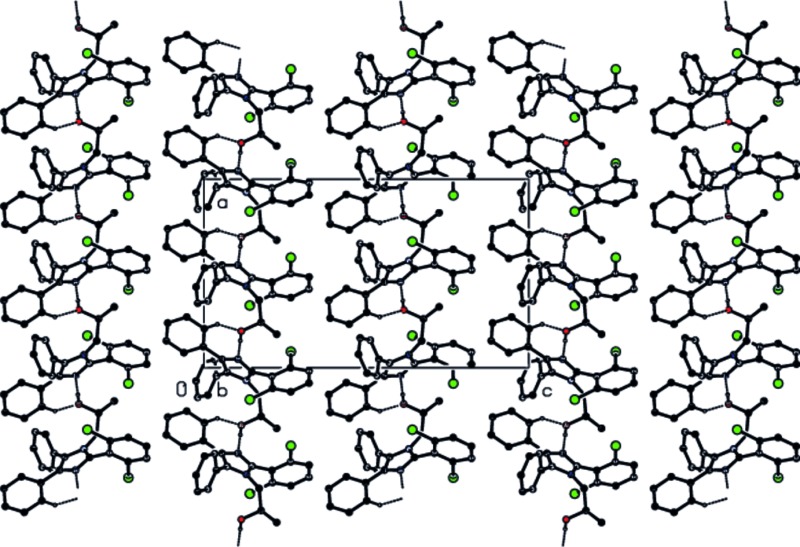
The molecular packing of the title compound viewed along the b axis. H atoms not involved in the hydrogen bonding (dashed lines) have been omitted for clarity. Only the major disorder component is shown.

. DOI: 10.1107/S2056989015006763/sj5450fig1.tif
Perspective view of the title mol­ecule with 30% probability displacement ellipsoids. Only the major disorder component is shown.

b . DOI: 10.1107/S2056989015006763/sj5450fig2.tif
The mol­ecular packing of the title compound viewed along the *b* axis. H atoms not involved in the hydrogen bonding (dashed lines) have been omitted for clarity. Only the major disorder component is shown.

CCDC reference: 1057878

Additional supporting information:  crystallographic information; 3D view; checkCIF report

## Figures and Tables

**Table 1:** Hydrogen-bond geometry (, ) *Cg*3 is the centroid of the C7C12 benzene ring.

*D*H*A*	*D*H	H*A*	*D* *A*	*D*H*A*
O1*B*H1*B*N2^i^	0.84	2.02	2.8468(1)	170
C14H14O1*B* ^ii^	0.95	2.42	3.2107(1)	141
C6*B*H6*B*2*Cg*3^iii^	0.98	2.78	3.6798(1)	154

Symmetry codes: (i) 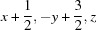; (ii) 
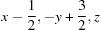
; (iii) 
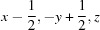
.

## supplementary crystallographic information

### S1. Comment

As part of an ongoing study on synthesis of imidazole based amino alcohols (Akkurt *et al.*, 2013; Mohamed *et al.*, 2013*a*,b; Jasinski *et al.*, 2015) we report herein the synthesis and crystal structure of the title compound, 1-[2-(2,6-dichlorophenyl)-4,5-diphenyl-1*H*-imidazol-1-yl] propan-2-ol.

In the title compound, Fig. 1, the central 1*H*-imidazole ring (N1B/N2/C1—C3) is twisted with respect to with the planes of the benzene, C7–C12, and two phenyl, C13–C18 and C19–C24, rings, making dihedral angles of 74.06 (18), 28.52 (17) and 67.65 (18)°, respectively. The dihedral angle between the C13–C18 and C19–C24 phenyl rings is 69.15 (18)°. The C7–C12 benzene ring makes dihedral angles of 87.01 (18) and 52.65 (18)° with the phenyl rings, C13–C18 and C19–C24, respectively. The bond lengths are normal and comparable to those reported for similar compounds (Mohamed *et al.*, 2012, 2013*a*,b; Akkurt *et al.*, 2013; Jasinski *et al.*, 2015).

In the crystal, O—H···N and C—H···O hydrogen bonds (Table 1) link molecules into chains along the *a* axis direction and the series of parallel chains displayed in Fig. 2 form a two-dimensional sheet approximately parallel to the *bc* diagonal. C—H···π interactions (Table 1) are also observed in the packing of the title compound.

### S2. Experimental

The title compound has been prepared according to our reported method (Jasinski *et al.*, 2015). Irregular colourless blocks of (I) were obtained by the slow evaporation method using ethanol as a solvent. *M*.p. 455 K, yield, 94%.

### S3. Refinement

The hydrogen atoms bonded to carbon atoms were located in geometrically idealized positions and constrained to ride on their parent atoms, with C—H = 0.95 - 1.00 Å, and with *U*_iso_(H) = 1.5*U*_eq_(C) for methyl H atoms and 1.2*U*_eq_(C) for other H atoms. The hydroxyl H atoms were found in difference Fourier maps and were constrained with O—H = 0.82 ± 0.02 Å and U(H) = 1.5 *U*_eq_(O). The N1 atom of the 1*H*-imidazole ring and the atoms (C4, C5, C6, O1) of the N1-bonded 2-hydroxypropyl group are disordered over two positions in a 0.722 (5):0.278 (5) ratio (N1A, N1B, C4A, C4B, C5A, C5B, C6A, C6B, O1A and O1B). Each pair of atoms in the disordered components N1A, N1B; C4A, C4B; C5A, C5B; C6A, C6B; O1A, O1B were constrained to be equal using the EADP instruction. In addition, the N1A and N1B atoms were set to occupy the same position by an EXYZ command. The (14 1 8), (14 0 - 4), (12 2 - 1), (13 1 3), (4 3 - 2), (14 2 - 4), (13 2 11), (12 3 - 2), (12 5 - 2), (11 6 4), (11 2 0) and (10 7 3) reflections were omitted owing to very bad agreement.

### Crystal data

**Table:**

C_24_H_20_Cl_2_N_2_O	*F*(000) = 880
*M**_r_* = 423.32	*D*_x_ = 1.311 Mg m^−^^3^
Orthorhombic, *P**n**a*2_1_	Cu *K*α radiation, λ = 1.54184 Å
Hall symbol: P 2c -2n	Cell parameters from 7461 reflections
*a* = 12.1468 (4) Å	θ = 4.2–71.3°
*b* = 8.4194 (2) Å	µ = 2.86 mm^−^^1^
*c* = 20.9636 (7) Å	*T* = 173 K
*V* = 2143.92 (11) Å^3^	Irregular blocks, colourless
*Z* = 4	0.48 × 0.44 × 0.26 mm

### Data collection

**Table:**

Agilent Eos Gemini diffractometer	4076 independent reflections
Radiation source: Enhance (Cu) X-ray Source	3888 reflections with *I* > 2σ(*I*)
Graphite monochromator	*R*_int_ = 0.033
Detector resolution: 16.0416 pixels mm^-1^	θ_max_ = 71.5°, θ_min_ = 4.2°
ω scans	*h* = −14→10
Absorption correction: multi-scan (*CrysAlis PRO*; Agilent, 2014)	*k* = −10→10
*T*_min_ = 0.541, *T*_max_ = 1.000	*l* = −25→25
16532 measured reflections	

### Refinement

**Table:**

Refinement on *F*^2^	Hydrogen site location: mixed
Least-squares matrix: full	H-atom parameters constrained
*R*[*F*^2^ > 2σ(*F*^2^)] = 0.037	*w* = 1/[σ^2^(*F*_o_^2^) + (0.0545*P*)^2^ + 0.7142*P*] where *P* = (*F*_o_^2^ + 2*F*_c_^2^)/3
*wR*(*F*^2^) = 0.097	(Δ/σ)_max_ < 0.001
*S* = 1.05	Δρ_max_ = 0.26 e Å^−^^3^
4076 reflections	Δρ_min_ = −0.18 e Å^−^^3^
278 parameters	Absolute structure: Refined as an inversion twin.
15 restraints	Absolute structure parameter: 0.068 (18)

### Special details

**Table:**

Geometry. All e.s.d.'s (except the e.s.d. in the dihedral angle between two l.s. planes) are estimated using the full covariance matrix. The cell e.s.d.'s are taken into account individually in the estimation of e.s.d.'s in distances, angles and torsion angles; correlations between e.s.d.'s in cell parameters are only used when they are defined by crystal symmetry. An approximate (isotropic) treatment of cell e.s.d.'s is used for estimating e.s.d.'s involving l.s. planes.
Refinement. Refined as a 2-component inversion twin.

### Fractional atomic coordinates and isotropic or equivalent isotropic displacement parameters (Å^2^)

**Table:**

	*x*	*y*	*z*	*U*_iso_*/*U*_eq_	Occ. (<1)
C1	0.0230 (2)	0.8162 (4)	0.64803 (16)	0.0295 (6)	
C2	−0.0445 (3)	0.8711 (4)	0.55579 (15)	0.0277 (6)	
C3	0.0249 (2)	0.9942 (4)	0.57117 (15)	0.0268 (6)	
C4A	0.141 (2)	1.064 (11)	0.667 (5)	0.030 (3)	0.278 (5)
H4A1	0.1256	1.0518	0.7132	0.036*	0.278 (5)
H4A2	0.1251	1.1758	0.6553	0.036*	0.278 (5)
C5A	0.2606 (16)	1.0292 (18)	0.6547 (8)	0.0314 (11)	0.278 (5)
H5A	0.2762	1.0391	0.6080	0.038*	0.278 (5)
C6A	0.3335 (15)	1.144 (3)	0.6916 (10)	0.057 (2)	0.278 (5)
H6A1	0.3217	1.2524	0.6758	0.086*	0.278 (5)
H6A2	0.4109	1.1148	0.6858	0.086*	0.278 (5)
H6A3	0.3148	1.1395	0.7370	0.086*	0.278 (5)
N1A	0.0677 (2)	0.9581 (3)	0.63059 (12)	0.0271 (5)	0.278 (5)
O1A	0.2788 (7)	0.8724 (11)	0.6741 (4)	0.0378 (7)	0.278 (5)
H1A	0.3136	0.8178	0.6509	0.057*	0.278 (5)
C4B	0.1417 (10)	1.055 (4)	0.6701 (19)	0.030 (3)	0.722 (5)
H4B1	0.1098	1.0639	0.7134	0.036*	0.722 (5)
H4B2	0.1450	1.1634	0.6518	0.036*	0.722 (5)
C5B	0.2591 (5)	0.9909 (7)	0.6759 (3)	0.0314 (11)	0.722 (5)
H5B	0.2570	0.8779	0.6905	0.038*	0.722 (5)
C6B	0.3226 (5)	1.0906 (10)	0.7239 (4)	0.057 (2)	0.722 (5)
H6B1	0.3215	1.2022	0.7105	0.086*	0.722 (5)
H6B2	0.3989	1.0533	0.7261	0.086*	0.722 (5)
H6B3	0.2882	1.0807	0.7660	0.086*	0.722 (5)
N1B	0.0677 (2)	0.9581 (3)	0.63059 (12)	0.0271 (5)	0.722 (5)
O1B	0.3065 (3)	0.9989 (4)	0.61501 (16)	0.0378 (7)	0.722 (5)
H1B	0.3568	0.9308	0.6120	0.057*	0.722 (5)
C7	0.0459 (3)	0.7343 (4)	0.70923 (16)	0.0314 (7)	
C8	0.0008 (3)	0.7834 (4)	0.76676 (18)	0.0380 (7)	
C9	0.0182 (3)	0.7043 (5)	0.82364 (19)	0.0491 (9)	
H9	−0.0131	0.7426	0.8622	0.059*	
C10	0.0821 (4)	0.5684 (5)	0.8233 (2)	0.0505 (10)	
H10	0.0943	0.5120	0.8619	0.061*	
C11	0.1281 (3)	0.5144 (4)	0.7672 (2)	0.0417 (8)	
H11	0.1719	0.4210	0.7672	0.050*	
C12	0.1104 (3)	0.5963 (4)	0.71118 (16)	0.0341 (7)	
C13	−0.1113 (3)	0.8452 (3)	0.49796 (15)	0.0277 (6)	
C14	−0.2071 (3)	0.7556 (4)	0.50122 (18)	0.0333 (7)	
H14	−0.2307	0.7153	0.5413	0.040*	
C15	−0.2688 (3)	0.7238 (4)	0.44710 (19)	0.0383 (8)	
H15	−0.3336	0.6612	0.4505	0.046*	
C16	−0.2374 (3)	0.7820 (5)	0.38861 (18)	0.0437 (8)	
H16	−0.2798	0.7596	0.3516	0.052*	
C17	−0.1426 (3)	0.8741 (5)	0.38446 (18)	0.0438 (9)	
H17	−0.1207	0.9164	0.3444	0.053*	
C18	−0.0797 (3)	0.9048 (4)	0.43837 (16)	0.0351 (7)	
H18	−0.0146	0.9667	0.4348	0.042*	
C19	0.0444 (3)	1.1482 (4)	0.53852 (15)	0.0287 (6)	
C20	−0.0426 (3)	1.2530 (4)	0.53319 (17)	0.0352 (7)	
H20	−0.1120	1.2261	0.5510	0.042*	
C21	−0.0295 (3)	1.3975 (4)	0.50194 (19)	0.0428 (8)	
H21	−0.0895	1.4694	0.4987	0.051*	
C22	0.0711 (4)	1.4357 (4)	0.47581 (19)	0.0447 (9)	
H22	0.0800	1.5338	0.4540	0.054*	
C23	0.1579 (3)	1.3342 (5)	0.4809 (2)	0.0479 (10)	
H23	0.2272	1.3625	0.4632	0.057*	
C24	0.1457 (3)	1.1879 (4)	0.51228 (18)	0.0395 (8)	
H24	0.2061	1.1167	0.5155	0.047*	
N2	−0.0448 (2)	0.7612 (3)	0.60455 (13)	0.0300 (6)	
Cl1	−0.08462 (8)	0.95060 (12)	0.76765 (5)	0.0517 (3)	
Cl2	0.16981 (8)	0.52689 (10)	0.64134 (5)	0.0454 (2)	

### Atomic displacement parameters (Å^2^)

**Table:**

	*U*^11^	*U*^22^	*U*^33^	*U*^12^	*U*^13^	*U*^23^
C1	0.0281 (15)	0.0269 (14)	0.0334 (15)	−0.0003 (11)	−0.0003 (13)	0.0026 (13)
C2	0.0268 (15)	0.0253 (14)	0.0310 (15)	0.0017 (11)	0.0014 (12)	0.0036 (12)
C3	0.0260 (15)	0.0231 (13)	0.0312 (15)	0.0015 (11)	0.0009 (12)	0.0017 (11)
C4A	0.0314 (17)	0.028 (4)	0.031 (4)	−0.0008 (14)	−0.0007 (14)	−0.005 (4)
C5A	0.037 (2)	0.030 (3)	0.027 (3)	0.003 (2)	−0.003 (3)	−0.004 (2)
C6A	0.037 (3)	0.078 (5)	0.056 (4)	0.011 (3)	−0.012 (3)	−0.034 (4)
N1A	0.0272 (13)	0.0249 (12)	0.0291 (14)	−0.0018 (10)	−0.0007 (10)	−0.0006 (9)
O1A	0.0399 (17)	0.0390 (18)	0.0345 (16)	0.0115 (13)	0.0053 (14)	0.0003 (13)
C4B	0.0314 (17)	0.028 (4)	0.031 (4)	−0.0008 (14)	−0.0007 (14)	−0.005 (4)
C5B	0.037 (2)	0.030 (3)	0.027 (3)	0.003 (2)	−0.003 (3)	−0.004 (2)
C6B	0.037 (3)	0.078 (5)	0.056 (4)	0.011 (3)	−0.012 (3)	−0.034 (4)
N1B	0.0272 (13)	0.0249 (12)	0.0291 (14)	−0.0018 (10)	−0.0007 (10)	−0.0006 (9)
O1B	0.0399 (17)	0.0390 (18)	0.0345 (16)	0.0115 (13)	0.0053 (14)	0.0003 (13)
C7	0.0302 (16)	0.0327 (16)	0.0313 (15)	−0.0045 (13)	−0.0005 (12)	0.0063 (13)
C8	0.0334 (17)	0.0409 (18)	0.0397 (17)	0.0031 (14)	0.0004 (15)	0.0058 (15)
C9	0.055 (2)	0.061 (2)	0.0318 (17)	0.004 (2)	0.0061 (17)	0.0057 (17)
C10	0.058 (2)	0.057 (2)	0.036 (2)	0.002 (2)	−0.0035 (17)	0.0211 (18)
C11	0.0415 (19)	0.0391 (18)	0.0446 (19)	0.0017 (15)	−0.0024 (17)	0.0121 (16)
C12	0.0340 (17)	0.0329 (16)	0.0355 (18)	−0.0038 (14)	0.0000 (14)	0.0046 (14)
C13	0.0290 (15)	0.0204 (13)	0.0339 (16)	0.0038 (11)	−0.0016 (12)	−0.0001 (12)
C14	0.0337 (17)	0.0268 (15)	0.0393 (17)	0.0011 (13)	−0.0016 (14)	0.0041 (13)
C15	0.0308 (16)	0.0301 (17)	0.054 (2)	−0.0025 (14)	−0.0097 (15)	0.0002 (15)
C16	0.047 (2)	0.0452 (19)	0.0389 (19)	0.0057 (16)	−0.0130 (16)	−0.0091 (15)
C17	0.048 (2)	0.053 (2)	0.0306 (18)	0.0016 (17)	−0.0005 (15)	−0.0008 (16)
C18	0.0340 (18)	0.0373 (17)	0.0339 (17)	0.0003 (14)	0.0011 (13)	0.0025 (14)
C19	0.0353 (17)	0.0216 (14)	0.0292 (15)	−0.0027 (12)	−0.0020 (12)	−0.0003 (12)
C20	0.0369 (18)	0.0308 (16)	0.0378 (17)	0.0030 (13)	0.0021 (15)	0.0044 (13)
C21	0.056 (2)	0.0255 (16)	0.047 (2)	0.0066 (15)	−0.0072 (17)	0.0030 (15)
C22	0.062 (3)	0.0271 (17)	0.045 (2)	−0.0137 (16)	−0.0128 (17)	0.0107 (15)
C23	0.041 (2)	0.050 (2)	0.053 (2)	−0.0220 (18)	−0.0037 (17)	0.0169 (18)
C24	0.0332 (18)	0.0364 (18)	0.049 (2)	−0.0033 (14)	−0.0011 (15)	0.0087 (16)
N2	0.0269 (13)	0.0278 (13)	0.0353 (14)	−0.0028 (10)	−0.0018 (11)	0.0046 (11)
Cl1	0.0463 (5)	0.0579 (5)	0.0509 (5)	0.0193 (4)	0.0084 (4)	0.0049 (4)
Cl2	0.0586 (5)	0.0359 (4)	0.0416 (4)	0.0092 (4)	0.0054 (4)	0.0020 (4)

### Geometric parameters (Å, º)

**Table:**

C1—N2	1.313 (4)	C8—C9	1.382 (5)
C1—N1A	1.362 (4)	C8—Cl1	1.749 (4)
C1—C7	1.483 (4)	C9—C10	1.382 (6)
C2—C3	1.374 (4)	C9—H9	0.9500
C2—N2	1.379 (4)	C10—C11	1.378 (6)
C2—C13	1.475 (4)	C10—H10	0.9500
C3—N1A	1.384 (4)	C11—C12	1.379 (5)
C3—C19	1.485 (4)	C11—H11	0.9500
C4A—N1A	1.47 (3)	C12—Cl2	1.734 (4)
C4A—C5A	1.51 (3)	C13—C14	1.388 (5)
C4A—H4A1	0.9900	C13—C18	1.400 (5)
C4A—H4A2	0.9900	C14—C15	1.386 (5)
C5A—O1A	1.399 (15)	C14—H14	0.9500
C5A—C6A	1.52 (2)	C15—C16	1.374 (6)
C5A—H5A	1.0000	C15—H15	0.9500
C6A—H6A1	0.9800	C16—C17	1.391 (6)
C6A—H6A2	0.9800	C16—H16	0.9500
C6A—H6A3	0.9800	C17—C18	1.388 (5)
O1A—H1A	0.7924	C17—H17	0.9500
C4B—C5B	1.530 (17)	C18—H18	0.9500
C4B—H4B1	0.9900	C19—C20	1.381 (5)
C4B—H4B2	0.9900	C19—C24	1.388 (5)
C5B—O1B	1.401 (6)	C20—C21	1.391 (5)
C5B—C6B	1.521 (7)	C20—H20	0.9500
C5B—H5B	1.0000	C21—C22	1.377 (6)
C6B—H6B1	0.9800	C21—H21	0.9500
C6B—H6B2	0.9800	C22—C23	1.362 (6)
C6B—H6B3	0.9800	C22—H22	0.9500
O1B—H1B	0.8400	C23—C24	1.404 (5)
C7—C8	1.388 (5)	C23—H23	0.9500
C7—C12	1.402 (5)	C24—H24	0.9500
			
N2—C1—N1A	111.9 (3)	C9—C8—Cl1	118.0 (3)
N2—C1—C7	123.7 (3)	C7—C8—Cl1	118.9 (3)
N1A—C1—C7	124.4 (3)	C8—C9—C10	118.7 (4)
C3—C2—N2	109.5 (3)	C8—C9—H9	120.7
C3—C2—C13	130.0 (3)	C10—C9—H9	120.7
N2—C2—C13	120.5 (3)	C11—C10—C9	120.3 (3)
C2—C3—N1A	106.0 (3)	C11—C10—H10	119.8
C2—C3—C19	130.4 (3)	C9—C10—H10	119.8
N1A—C3—C19	123.2 (3)	C10—C11—C12	119.9 (3)
N1A—C4A—C5A	112 (2)	C10—C11—H11	120.1
N1A—C4A—H4A1	109.2	C12—C11—H11	120.1
C5A—C4A—H4A1	109.2	C11—C12—C7	121.8 (3)
N1A—C4A—H4A2	109.2	C11—C12—Cl2	119.1 (3)
C5A—C4A—H4A2	109.2	C7—C12—Cl2	119.2 (2)
H4A1—C4A—H4A2	107.9	C14—C13—C18	117.9 (3)
O1A—C5A—C4A	107 (5)	C14—C13—C2	120.1 (3)
O1A—C5A—C6A	111.1 (14)	C18—C13—C2	122.0 (3)
C4A—C5A—C6A	110.4 (17)	C15—C14—C13	121.2 (3)
O1A—C5A—H5A	109.6	C15—C14—H14	119.4
C4A—C5A—H5A	109.6	C13—C14—H14	119.4
C6A—C5A—H5A	109.6	C16—C15—C14	120.7 (3)
C5A—C6A—H6A1	109.5	C16—C15—H15	119.6
C5A—C6A—H6A2	109.5	C14—C15—H15	119.6
H6A1—C6A—H6A2	109.5	C15—C16—C17	119.0 (3)
C5A—C6A—H6A3	109.5	C15—C16—H16	120.5
H6A1—C6A—H6A3	109.5	C17—C16—H16	120.5
H6A2—C6A—H6A3	109.5	C18—C17—C16	120.6 (3)
C1—N1A—C3	106.5 (3)	C18—C17—H17	119.7
C1—N1A—C4A	129 (5)	C16—C17—H17	119.7
C3—N1A—C4A	124 (5)	C17—C18—C13	120.6 (3)
C5A—O1A—H1A	116.9	C17—C18—H18	119.7
C5B—C4B—H4B1	108.6	C13—C18—H18	119.7
C5B—C4B—H4B2	108.6	C20—C19—C24	119.5 (3)
H4B1—C4B—H4B2	107.6	C20—C19—C3	118.2 (3)
O1B—C5B—C6B	111.6 (5)	C24—C19—C3	122.3 (3)
O1B—C5B—C4B	107.1 (17)	C19—C20—C21	120.6 (3)
C6B—C5B—C4B	109.3 (6)	C19—C20—H20	119.7
O1B—C5B—H5B	109.6	C21—C20—H20	119.7
C6B—C5B—H5B	109.6	C22—C21—C20	119.6 (3)
C4B—C5B—H5B	109.6	C22—C21—H21	120.2
C5B—C6B—H6B1	109.5	C20—C21—H21	120.2
C5B—C6B—H6B2	109.5	C23—C22—C21	120.6 (3)
H6B1—C6B—H6B2	109.5	C23—C22—H22	119.7
C5B—C6B—H6B3	109.5	C21—C22—H22	119.7
H6B1—C6B—H6B3	109.5	C22—C23—C24	120.3 (4)
H6B2—C6B—H6B3	109.5	C22—C23—H23	119.8
C5B—O1B—H1B	109.5	C24—C23—H23	119.8
C8—C7—C12	116.2 (3)	C19—C24—C23	119.4 (3)
C8—C7—C1	122.6 (3)	C19—C24—H24	120.3
C12—C7—C1	121.0 (3)	C23—C24—H24	120.3
C9—C8—C7	123.1 (3)	C1—N2—C2	106.0 (3)
			
N2—C2—C3—N1A	0.0 (3)	C1—C7—C12—C11	−176.8 (3)
C13—C2—C3—N1A	−179.2 (3)	C8—C7—C12—Cl2	180.0 (3)
N2—C2—C3—C19	−172.4 (3)	C1—C7—C12—Cl2	3.4 (4)
C13—C2—C3—C19	8.4 (6)	C3—C2—C13—C14	−152.9 (3)
N1A—C4A—C5A—O1A	−61 (9)	N2—C2—C13—C14	28.0 (4)
N1A—C4A—C5A—C6A	178 (6)	C3—C2—C13—C18	28.9 (5)
N2—C1—N1A—C3	−0.4 (4)	N2—C2—C13—C18	−150.2 (3)
C7—C1—N1A—C3	−179.3 (3)	C18—C13—C14—C15	0.9 (5)
N2—C1—N1A—C4A	175.3 (18)	C2—C13—C14—C15	−177.3 (3)
C7—C1—N1A—C4A	−3.6 (19)	C13—C14—C15—C16	−0.7 (5)
C2—C3—N1A—C1	0.2 (3)	C14—C15—C16—C17	−0.3 (6)
C19—C3—N1A—C1	173.3 (3)	C15—C16—C17—C18	1.0 (6)
C2—C3—N1A—C4A	−175.8 (18)	C16—C17—C18—C13	−0.8 (6)
C19—C3—N1A—C4A	−2.7 (19)	C14—C13—C18—C17	−0.2 (5)
C5A—C4A—N1A—C1	92 (8)	C2—C13—C18—C17	178.0 (3)
C5A—C4A—N1A—C3	−93 (8)	C2—C3—C19—C20	62.2 (5)
N2—C1—C7—C8	−103.3 (4)	N1A—C3—C19—C20	−109.1 (4)
N1A—C1—C7—C8	75.4 (5)	C2—C3—C19—C24	−116.4 (4)
N2—C1—C7—C12	73.1 (4)	N1A—C3—C19—C24	72.3 (4)
N1A—C1—C7—C12	−108.2 (4)	C24—C19—C20—C21	−0.1 (5)
C12—C7—C8—C9	0.9 (5)	C3—C19—C20—C21	−178.7 (3)
C1—C7—C8—C9	177.4 (3)	C19—C20—C21—C22	0.4 (6)
C12—C7—C8—Cl1	−177.8 (3)	C20—C21—C22—C23	−0.8 (6)
C1—C7—C8—Cl1	−1.2 (5)	C21—C22—C23—C24	0.8 (6)
C7—C8—C9—C10	−1.1 (6)	C20—C19—C24—C23	0.2 (5)
Cl1—C8—C9—C10	177.6 (3)	C3—C19—C24—C23	178.7 (3)
C8—C9—C10—C11	0.6 (6)	C22—C23—C24—C19	−0.5 (6)
C9—C10—C11—C12	0.0 (6)	N1A—C1—N2—C2	0.4 (4)
C10—C11—C12—C7	−0.2 (6)	C7—C1—N2—C2	179.3 (3)
C10—C11—C12—Cl2	179.6 (3)	C3—C2—N2—C1	−0.2 (4)
C8—C7—C12—C11	−0.2 (5)	C13—C2—N2—C1	179.0 (3)

### Hydrogen-bond geometry (Å, º)

Cg3 is the centroid of the C7–C12 benzene ring.

**Table:**

*D*—H···*A*	*D*—H	H···*A*	*D*···*A*	*D*—H···*A*
O1*B*—H1*B*···N2^i^	0.84	2.02	2.8468 (1)	170
C4*B*—H4*B*1···Cl1	0.99	2.79	3.5382 (1)	133
C14—H14···O1*B*^ii^	0.95	2.42	3.2107 (1)	141
C6*B*—H6*B*2···*Cg*3^iii^	0.98	2.78	3.6798 (1)	154

Symmetry codes: (i) *x*+1/2, −*y*+3/2, *z*; (ii) *x*−1/2, −*y*+3/2, *z*; (iii) *x*−1/2, −*y*+1/2, *z*.
