# Supplementary figures and images for: Crystal structure of 1-[2-(2,6-di­chloro­phen­yl)-4,5-diphenyl-1H-imidazol-1-yl]propan-2-ol
Source: Acta Crystallogr E Crystallogr Commun. 2015 Apr 9;71(Pt 5):o299–300. doi: 10.1107/S2056989015006763 (PMC4420112; doi:10.1107/S2056989015006763)

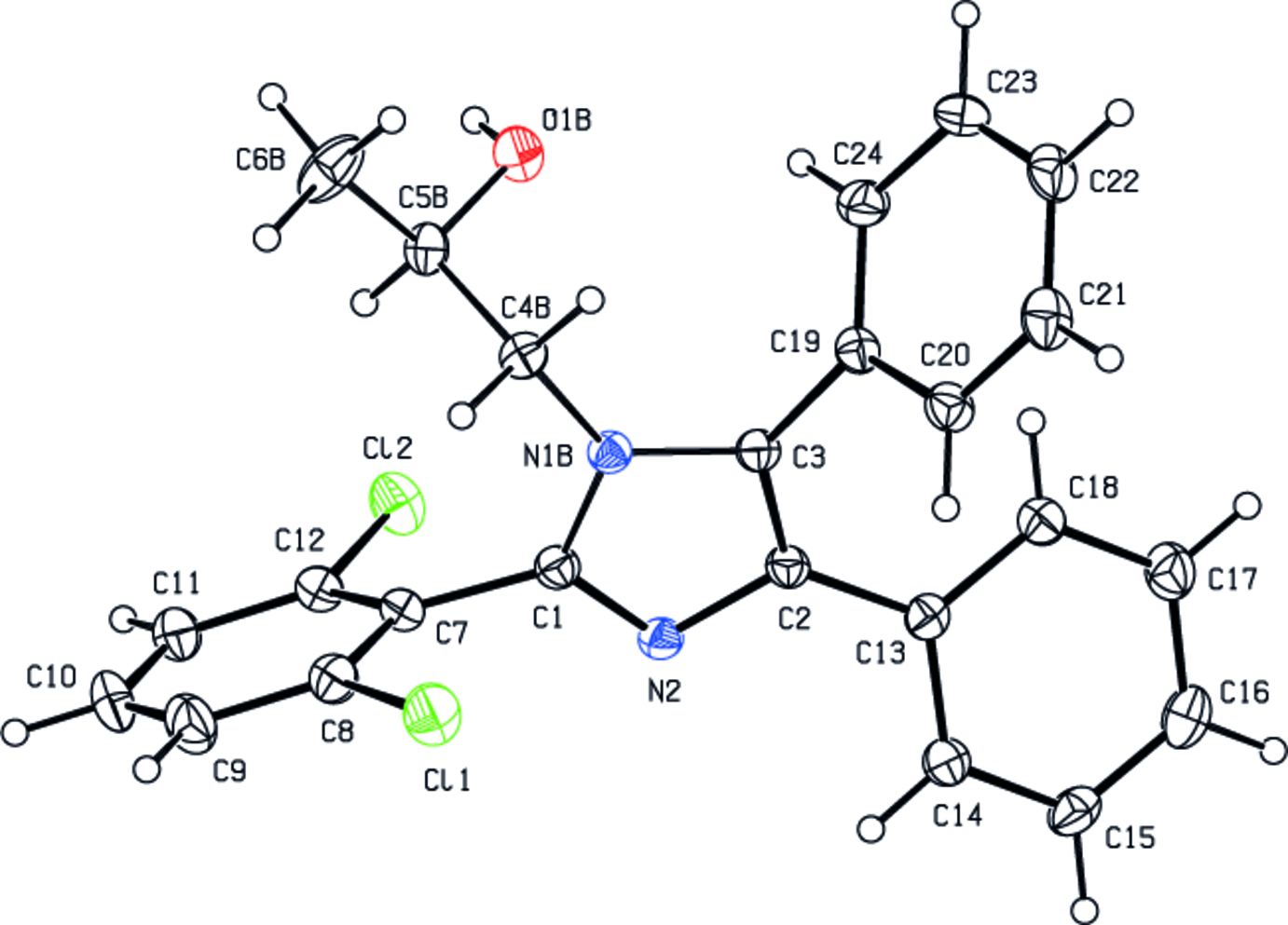

Supplement: Supplementary file 4 [file e-71-0o299-fig1.tif]

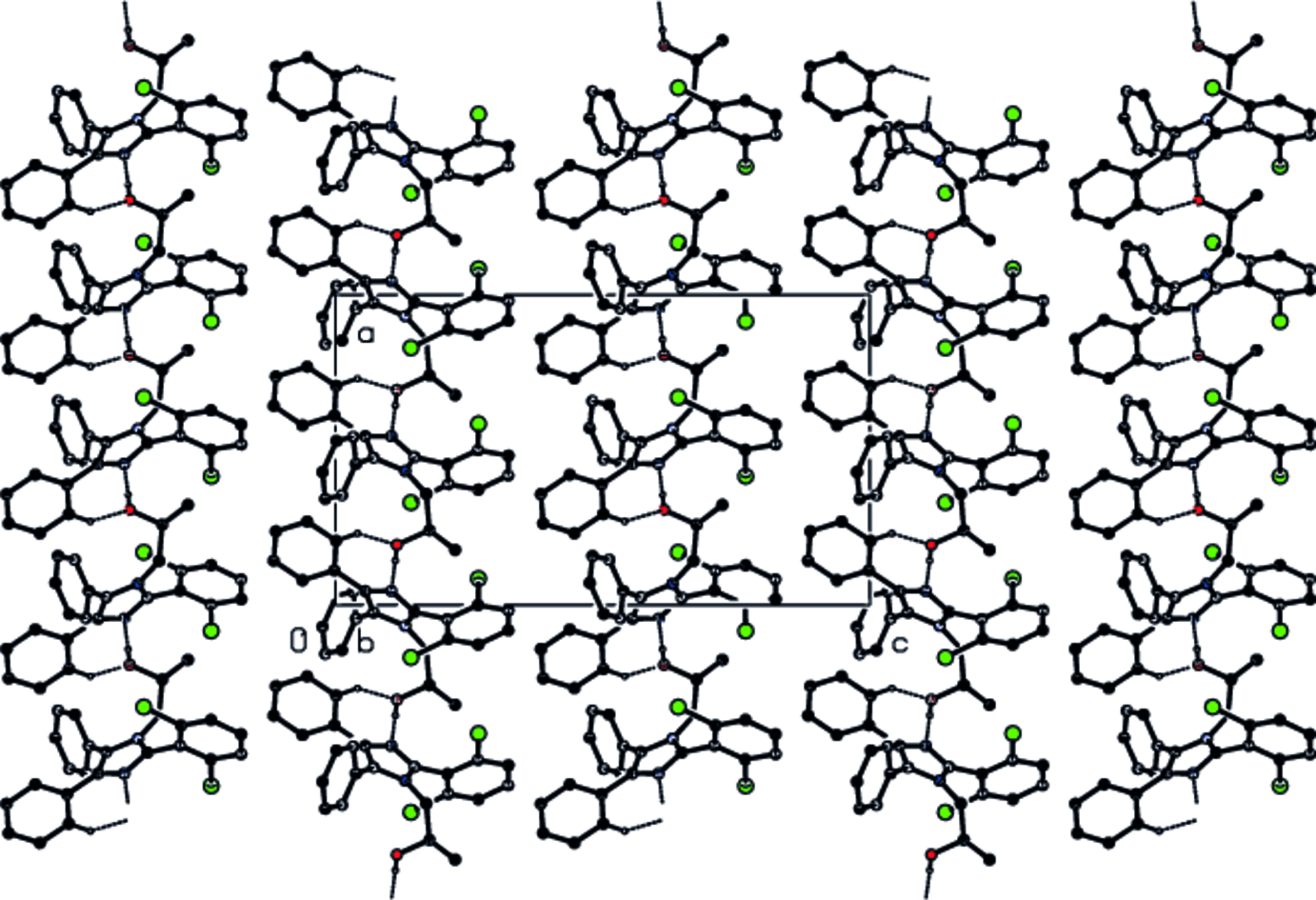

Supplement: Supplementary file 5 [file e-71-0o299-fig2.tif]
